# Coordinated inauthentic behavior: An innovative manipulation tactic to amplify COVID-19 anti-vaccine communication outreach *via* social media

**DOI:** 10.3389/fsoc.2023.1141416

**Published:** 2023-03-16

**Authors:** Monica Murero

**Affiliations:** Department of Social Sciences, University of Naples Federico II, Naples, Italy

**Keywords:** coordinated inauthentic behavior, digital society, online manipulation, COVID-19 anti-vaccine debate, deceived social media algorithms, technology exploitation, online harmful behavior, authenticity of online public debate

## Abstract

Coordinated inauthentic behavior (CIB) is a manipulative communication tactic that uses a mix of authentic, fake, and duplicated social media accounts to operate as an adversarial network (AN) across multiple social media platforms. The article aims to clarify how CIB's emerging communication tactic “secretly” exploits technology to massively harass, harm, or mislead the online debate around crucial issues for society, like the COVID-19 vaccination. CIB's manipulative operations could be one of the greatest threats to freedom of expression and democracy in our society. CIB campaigns mislead others by acting with pre-arranged exceptional similarity and “secret” operations. Previous theoretical frameworks failed to evaluate the role of CIB on vaccination attitudes and behavior. In light of recent international and interdisciplinary CIB research, this study critically analyzes the case of a COVID-19 anti-vaccine adversarial network removed from Meta at the end of 2021 for *brigading*. A violent and harmful attempt to tactically manipulate the COVID-19 vaccine debate in Italy, France, and Germany. The following focal issues are discussed: (1) CIB manipulative operations, (2) their extensions, and (3) challenges in CIB's identification. The article shows that CIB acts in three domains: (i) structuring inauthentic online communities, (ii) exploiting social media technology, and (iii) deceiving algorithms to extend communication outreach to unaware social media users, a matter of concern for the general audience of CIB-illiterates. Upcoming threats, open issues, and future research directions are discussed.

## 1. Introduction

Coordinated Inauthentic Behavior (CIB) is an emerging manipulative communication tactic using a mix of authentic, *fake*, and duplicated social media *accounts* to operate as an adversarial network (AN) across multiple social media platforms. This article aims to clarify how CIB's emerging communication tactic “secretly” exploits social media technology to massively harass, harm, and mislead the online debate around crucial issues for society, like the COVID-19 vaccination.

Hiding the real identities of the *adversarial network's* leaders is part of the CIB deception. CIB campaigns mislead others about who they are and what they are doing by acting with pre-arranged or exceptional similarities (Cinelli et al., 2022). In late 2021, Meta removed an extensive anti-vaccine *adversarial network*, violently acting across multiple social media platforms to oppose COVID-19 vaccination (Gleicher et al., 2021; Graphika, 2021).

Hard-to-detect CIBs' manipulative operations, massively spreading disinformation, and attacking *unaware* targets and opponents could be one of the greatest threats to freedom of expression and democracy in the highly populated social media ecosystem (Woolley and Howard, 2016; Vo et al., 2017; Howard et al., 2018; Peretti-Watel et al., 2020; Hristakieva et al., 2022; Mehta et al., 2022; Nguyen et al., 2022).

Understanding more deeply the CIB online dynamics, extension, and technological failures is very important since there is a concrete risk that coordinated, repetitive, and harmful efforts “invisibly” impact attitudes and decision-making on crucial issues for our society without the general audience's clear knowledge of the problem. Consequently, reviewing recent work concerning CIB manipulative communication tactics becomes highly relevant to social scientists and the general audience, politicians, and policymakers.

Since CIB is an emerging phenomenon, it is still unclear how it “secretly” *operates via* social media and to what *extent* malicious agents' inauthentic (harmful) coordination could harm democracy and society. Moreover, how CIBs are deliberately organized, resourced, and reinforced by digital platforms' algorithms and paid services is still unclear, a little-studied phenomenon in social sciences that needs further investigation.

In order to clarify these issues, this article is organized into three parts: first, traditional theoretical frameworks that may explain vaccination attitudes and behaviors are considered. Second, current interdisciplinary and international literature is reviewed according to three research questions and dimensions: (a) CIB operations (contents, agents), (b) expansion, and (c) identification (challenges). In this context, a critical examination emerges from analyzing a rare case of COVID-19 anti-vaccination network removed from Meta at the end of 2021. Third, open issues, current limits, and a future research agenda on CIB manipulative tactics are discussed.

### 1.1. Hypothesis and research questions

**Figure 1:**
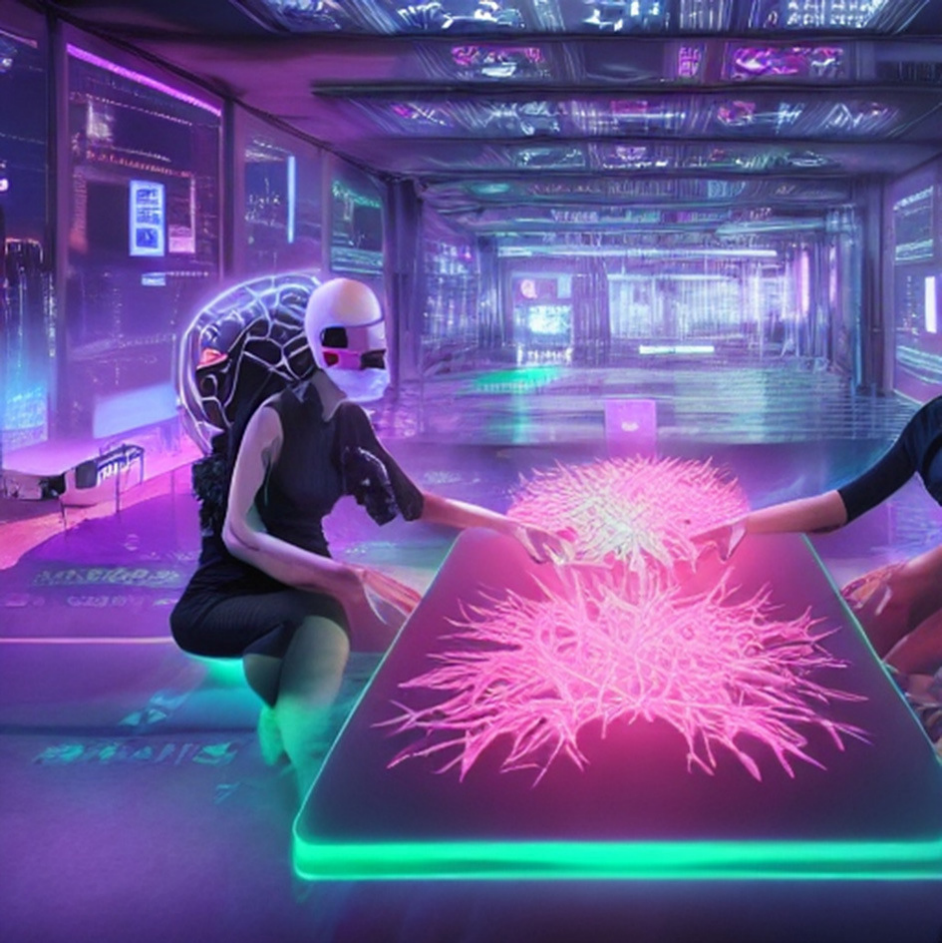
The image is AI generated using a text to Artificial Intelligence art generator technology from deepAI.org.

The central hypothesis that moves this study's investigation is that emerging forms of coordinated inauthentic behavior exploit *platforms'* technology (features, *logic*, and vulnerability) to deceive the public communication debate.^1^ Consequently, the research questions include the following:

(RQ1) How CIBs *operate*? Do CIB manipulative operations revolve around inauthentic content or malicious agents' tactics? (i.e., psychological intimidation, mass harassment, and harmful behavior).
(RQ2) Could social media technology *extend* CIB's manipulative communication outreach? (i.e., exploiting current features, popularity index *metrics*, and social media paid advertisement services). How CIBs deceive social media algorithms?
(RQ3) What are the challenges in *identifying* CIB in the vast social media ecosystem? (Figure 1).

## 2. Vaccination attitudes and behaviors: From traditional theories to CIB

Vaccine opposition, hesitancy, and acceptance (Kata, 2012; Xu and Guo, 2018; Eichhorn et al., 2022) have existed as long as vaccination itself (Durbach, 2000) and date back to 1796 when the British doctor, Edward Jenner (1749–1823) invented the first vaccine against viral disease (smallpox) in the UK.

Theoretical frameworks and constructs based on empirical observations of human argumentations emerged over time (Murero and Rice, 2006). However, previous research (Fishbein and Ajzen, 1975; Brehm and Brehm, 1981; Moscovici, 1987; Kunda, 1990; Goertzel, 1994; Bandura, 1997; Blume, 2006; Hobson-West, 2007; Lunt and Pantti, 2007; Gobo and Sena, 2019; Vochocová et al., 2022) shows that traditional sociological and cognitive theories have not yet sufficiently evaluated the *manipulative* role of CIB on COVID-19 vaccination attitudes and behaviors in contemporary *online* debates.

The theoretical gap could be due, in part, to the fact that CIB has appeared in the social media ecosystem only recently. Since the Cambridge Analytics scandal emerged,^2^ organized manipulative campaigns have been rising on digital platforms.

## 3. CIB operations and expansion

### 3.1. Coordinated content and fake news

In 2017, Meta introduced the term “Coordinated Inauthentic Behavior”.^3^ CIBs have grown exponentially, in the last five years. CIB *coordination* appeared as a deliberated and resourced orchestration of malicious actors tactically exploiting technology features to reach specific goals (Gleicher, 2018). In online manipulation studies, the term *coordination* is essential because reaching a certain number of social media users is crucial to obtaining a successful communication outreach (Cinelli et al., 2022). The second term, *inauthenticity*, is still under discussion in the literature (Szczesniak et al., 2020; Zinovyeva et al., 2020), although in association with *harmfulness* has a more distinctive connotation for the analysis of online manipulation exploiting contemporary technology.

Early research on CIB operations concentrated on two essential aspects: fake *content* and malicious *actors*. Initially, research focused on *inauthentic content* since the CIB's operations often correlate with misleading and fake news. In this context, the broad definition of “fake” and “news” (Caplan et al., 2018; HLEG EU Commission, 2018) encouraged the development of alternative terminologies to narrow the issue (Wardle and Derakhshan, 2017). For example, terms such as “fake news commercially motivated” (Silverman, 2017) have described CIB as aiming at economic fraud (Mazza et al., 2022). Other authors have *expanded* the meaning of the term “fake,” including problematic information (Jack, 2017), while others have looked at contact points among similar occurrences. Examples include fake news and propaganda (Giatsoglou et al., 2015; Vo et al., 2017), propaganda and misleading information (Hristakieva et al., 2022), *disinformation* (Del Vicario et al., 2016; Lazer et al., 2018; Zhang and Ghorbani, 2020), *misinformation* and *malinformation* (Dame Adjin-Tettey, 2022), harassment and hateful speech (Zinovyeva et al., 2020; Hristakieva et al., 2022), and personal opinions and radicalization (Santos et al., 2021).

### 3.2. Malicious agents: The case of COVID-19 anti-vaccine adversarial network

Over time, research on inauthentic and fake content pointed to a second danger: *malicious actors* (Borges do Nascimento et al., 2022; Dame Adjin-Tettey, 2022). Their coordinated efforts appeared to successfully spread inauthentic content by acting as an adversarial network (AN) harassing and intimidating the opponent's view.

In December 2021, a large adversarial network was removed from all Meta platforms (Gleicher et al., 2021) for *brigading*. Brigading activity “*can range from highly sophisticated intimidation operations to stifle dissent, to crude harassment campaigns to drown out opposing viewpoints”*.^4^

The network secretly coordinated repetitive operations, violently harassing and psychologically intimidating *supporters* of COVID-19 vaccination. The removed AN was interconnected to V_V, an anti-vaccination group that originated in Italy and France, which publicly reported engaging in violent online and offline coordinated operations.

CIB anti-vaccine malicious agents followed a structured *pyramidal hierarchy* of power, using a common language, and often adopted the same profile pictures within the group, coherently with previous research on manipulative networks' communication (Vo et al., 2017; Peretti-Watel et al., 2020). In this context, the AN removed by Meta appeared to rely on a structured combination of online agents (real, fake, and duplicated *accounts*) opposing what they called “the healthcare dictatorship” (Graphika, 2021, p. 5) and attacking the “Nazi supporters” (active vaccination promoters).

The leading actors used Telegram secret chat with self-destructing message features to spread orders and pre-arranged content, evade detection, and secretly train new members.^5^ Unlike their leaders, AN supporters often revealed their identity to promote genuine anti-vaccination active engagement in online and offline contexts, coherently with previous research (Hristakieva et al., 2022) on CIB proselytism.

### 3.3. CIB operations: Psycho-tech manipulation

The anti-vaccination network removed by Meta operated according to at least two manipulation tactics, often appearing together, to expand CIB's communication outreach: (1) psychological harassment and (2) exploitation of technology *logic* (van Dijck, 2013) and features.

According to Gleicher et al. (2021), the anti-vaccine network's members identified themselves as “*warriors”* fighting a *psychological* war against COVID-19 vaccination sustainers in Italy, France, Germany, and other countries. The anti-vaccine *leaders* coordinated actions across multiple digital platforms (Facebook, YouTube, Twitter, and VKontakte) to psychologically mass-harass specific targets in online and offline contexts. For example, the removed network aimed at misleading and silencing specific *targets* who had publicly sustained vaccination—journalists, health care providers, politicians, actors, and social media influencers.

The psychological fight involved sophisticated intimidation operations in repressing dissent and violent harassment campaigns to obscure opposing viewpoints. CIB's psychological harassment included a massive use of techno-features such as *dislike buttons* (to disapprove of pro-vaccine posts), orchestrated negative comments to opponent's *posts*, or even requests to “suspend” specific (pro-vaccine) accounts by inauthentically and massively reporting misconduct (Porreca et al., 2020). The “psychological war” extended to *offline target*s too. Examples of online–offline coordinated destructive campaigns included intimidation of doctors and vaccination hub vandalism. Moreover, people were encouraged to book vaccine appointments and not show up in the hope that unused doses would expire and be thrown out.^6^ Images of offline vandalic acts were posted to social media as “proof” of success to share with others and achieve rewards in terms of hierarchical advancements (Graphika, 2021, p. 21).

The second manipulative tactic, in part already emerged in the previous examples, focuses on CIB's ability to exploit social media *technology* to extend communication outreach, coherently with previous literature (Zhang and Ghorbani, 2020; Mehta et al., 2022). Malicious attempts to harm the online debate on COVID-19 vaccination showed *interdigital* (Murero, 2014, 2022) disinformation patterns. Inauthentic coordination aimed at reaching different goals and media to spread disinformation (Graphika, 2021, p. 13) by using pre-arranged content, hyperlinks (Giglietto et al., 2020; Santos et al., 2021), and identical blocks of hashtags.

Explicit knowledge of the digital platform's *logic* and weaknesses seems to represent an opportunity for manipulative communication outreach (Nguyen et al., 2022). For example, COVID-19 anti-vaccine campaigns were repetitively posted on Meta's popular pages *unrelated* to the vaccination debate (music and entertainment, pop culture, and food) to tactically gain *digital visibility* and therefore increase algorithmic “popularity” ranking metrics (Hristakieva et al., 2022).

### 3.4. The degree of extension of CIB

The strategic use of social media *connectivity* in popular pages unrelated to vaccination appeared to successfully outreach vast networks of subjects, beyond anti-vaccine groups, through personal ties among friends and family members (Howard et al., 2018). However, the degree of *extension* a CIB communication campaign can reach still needs to be clarified in the international literature. In recent studies (Giglietto et al., 2019; Mehta et al., 2022; Nguyen et al., 2022), the extent of CIB operations seems to dramatically change, depending on at least three aspects: (1) the sum of each malicious actor's “popularity metrics” on social media, (2) the available budget to invest in digital paid services (i.e., advertisement), and (3) the role of digital platforms' deceived algorithms.

First, the actor or account “popularity” is a measure estimated by the platform's algorithms (quantity of followers, shared content, views, likes, comments, and more). Individual metrics mainly depend on two aspects (a) the *extension* of the digital activity each account can reach and (b) the *reactions* of others, such as the number of comments a post reaches, the number of likes/dislikes obtained, the type, and the number of emoji reactions and more.

A second aspect influencing the degree of *extension* a CIB communication campaign can reach is economic. Social media offer a range of paid advertising services that users can purchase to promote their posts for specific micro-targeting actions beyond their followers' reach. Questionably, but coherently with the social media business *logic*, a significant investment could enormously extend the range of communication outreach.

### 3.5. Deceived algorithms and communication outreach

Adversarial network attacks using CIB have already exposed the vulnerability of social media platforms' ranking and recommendation algorithms, both theoretically and empirically. By ranking and recommending “interesting” (or sponsored) content to the platform's users, social media algorithms can significantly advantage those interested in amplifying visibility and information outreach. However, combining paid ads or *quantified attention* (Phillips, 2018) and viral content could *deceive* digital platform algorithms' ranking and recommendation systems. For example, when CIB misleading contents reach “good-performing” quantity attention criteria and become *popular* and “interesting” to others, then (deceived) algorithms could spread disinformation faster (Mehta et al., 2022). Therefore, there is a concrete risk that *deceived* algorithm technologies may fortify CIB's manipulative tactics and communication outreach.

## 4. Challenges in CIB identification (and removal)

After several months of malicious activity, Meta announced that an extensive anti-vaccine network, inauthentically behaving as a “brigade,” was *identified* and removed from all its platforms. Detecting CIB manipulative operations is challenging (Borges do Nascimento et al., 2022; Broniatowski et al., 2022; Curley et al., 2022) for digital platforms' security algorithms in the current scenario, where almost 5 billion people interact *via* social media.

In the last years, interdisciplinary studies have used sophisticated mixed methods (Amaturo and Punziano, 2021) to identify online communication issues, including artificial intelligence (Murero, 2020) to differentiate between authentic and inauthentic (manipulating) coordination of online campaigns (Vo et al., 2017; Jiang et al., 2021; Mazza et al., 2022).

The availability of adequate computational, economical, and human resources still limits rapid advances in CIB identification research.

Safeguarding the authenticity of the online debate is crucial for *unaware* social media users, the general audience, and society as a whole (Mazumdar and Thakker, 2020). Emerging sophisticated tactics may further threaten online debate's authenticity (Rao et al., 2021). Within this context, recent studies have shown that posting a substantial number of coordinated automated messages (i.e., *social bots*) can influence opinion trends (Howard et al., 2018) and amplify low-credibility information outreach (Shao et al., 2018). This evidence shows that recent CIB campaigns using a mix of artificial intelligence, innovative automated communication, and human-operated accounts (i.e., trolls) are becoming harder to identify (Boneh et al., 2019; Starbird, 2019). Social media corporations counter-measure CIB attacks on their platforms and remove emerging identified threats. However, it is still being determined to which extent digital platforms' security tactics are effective, over time, in protecting social media users' communication from CIB attacks since public access to private corporate platforms' security data is currently very limited (Broniatowski et al., 2022).

## 5. Final remarks

*Coordinated Inauthentic Behavior* is an innovative manipulation tactic that amplifies COVID-19 anti-vaccine communication outreach *via* social media. CIB has emerged as a worrisome and challenging phenomenon in three domains: (i) operative coordination, (ii) techno-manipulation extensibility, and (iii) identification/removal.

CIBs' manipulative communication could be one of the greatest threats to freedom of expression and democracy in the social media ecosystem (Woolley and Howard, 2016; Vo et al., 2017; Howard et al., 2018; Peretti-Watel et al., 2020; Hristakieva et al., 2022; Mehta et al., 2022; Nguyen et al., 2022). Recent evidence from the international and interdisciplinary research analyzed in this study clarified that *hard-to-identify* CIB manipulation could be dangerous to democracy and society because of how it is deliberately *organized*, resourced by malicious actors, and *reinforced* by digital technologies. CIBs mislead others, following manipulative goals and communication tactics, and hiding the identity of their leaders.

CIBs are a severe threat to unaware individuals interacting online, who might discuss public health policy issues with networked malicious agents, sharing pre-arranged content and disinformation rather than with genuine opponents, debating in a democratic scenario.

The removal from all Meta platforms of an extensive adversarial network *(brigading)* revealed a violent attempt to repetitively manipulate the COVID-19 vaccine debate in Italy, France, and Germany by exploiting current technology opportunities and weaknesses (Gleicher et al., 2021; Graphika, 2021). CIB operations are not limited to online digital environments but extend to society (home- harassment to intimidate *influencers*, medical professionals, and their family members). Sabotage of the vaccination hubs booking system resulted in economic damage, public health challenges, and an impediment to others willing to vaccinate, particularly fragile and older people.

We should note that Meta has not (yet) authorized this author's request to access (big) data from the removed anti-vaccine adversarial network, limiting the possibility of empirically analyzing the phenomenon. To overcome this limit, in the present article, CIB dynamics were observed in different contexts to identify emerging threats to online/offline communication internationally. The manipulative influence of CIBs is rapidly growing in political debates (Howard et al., 2018; Giglietto et al., 2019) and online economic frauds (Cinelli et al., 2022; Mazza et al., 2022). Research on CIB and public health issues is rare and should be further developed in different contexts. Responses to cope with this complexity and its effects are in urgent demand.

Future research should address the social implications of repetitive harmful CIB operations and their extensions in different social contexts over time, across multiple media, and on specific targets (particularly social media heavy users). Also, traditional theoretical frameworks explaining online vaccination issues (hesitancy, opposition, and acceptance) should evaluate the intervening role of CIB manipulative campaigns on individual attitudes and decision-making. The measurement of the impact of CIB on individuals and society could offer crucial evidence to understand manipulative tactics and counteract them in depth.

In an emerging digital environment where machines can generate text in online discussions, and people expect to interact with genuine opponents, this study suggests that communication campaigns of public health initiatives should consider more deeply the role of malicious agents' coordinated efforts in massively exploiting (still) unregulated sophisticated technology and outreach communication goals. Safeguarding the *authenticity* of the online debate and identifying emerging online/offline threats due to advances in sophisticated technologies like Artificial Intelligence (Hagen et al., 2020; Murero, 2020; Rao et al., 2021) is crucial not only for the online communication debate but also for the whole of society. Multi-platform interdisciplinary research, rapid identification of upcoming digital threats, and strict regulation involving stakeholders may help design innovative, private, and public policy responses that mitigate, instead of increase, the potential manipulative effect of CIB in the pandemic time and beyond.

## Author contributions

The author confirms being the sole contributor of this work and has approved it for publication.

## Footnotes

^1^These include but are not limited to organized tactics for psychologically silencing COVID-19 vaccination opponents and deceiving social media algorithms, online *anonymity* of perpetrators, and advertisement investments aimed at micro-targeting communication outreach.

^2^In 2018, when the Cambridge Analytics scandal emerged, concerns about the strategic use of online personal data, micro-targeting, and coordinated manipulation to influence attitudes and behaviors developed significantly, particularly in research analyzing CIB in online political debates and economic fraud.

^3^Meta security policy states, “We view CIB as coordinated efforts to manipulate public debate for a strategic goal where fake accounts are central to the operation”. There are two types of these activities that we work to stop: (1) coordinated inauthentic behavior in the context of domestic, non-government campaigns, and (2) coordinated inauthentic behavior on behalf of a foreign or government actor. When we find campaigns that include groups of accounts and Pages seeking to mislead people about who they are and what they are doing while relying on fake accounts, we remove both inauthentic and authentic accounts, Pages, and Groups directly involved in this activity. Source: Meta on CIB Report, October 2021. https://about.fb.com/wp-content/uploads/2021/11/October-2021-CIB-Report-Updated-Nov-5.pdf (accessed December 9, 2021).

^4^“We will remove any adversarial networks we find where people work together to mass comment, mass post, or engage in other types of repetitive mass behaviors to harass others or silence them”. Source Meta, https://about.fb.com/wp-content/uploads/2021/12/Metas-Adversarial-Threat-Report.pdf (accessed June 14 2022).

^5^Through videos, audio, and live interviews.

^6^For example, in Northern Italy, thousands of “warriors” booked their vaccination spot online and then did not show up to get vaccinated, resulting in deserted vaccination hubs, a significant economic waste, and an impediment to others willing to get vaccinated, particularly during the beginning of the COVID-19 vaccination campaign, in 2021.
